# Identification of the Interactors of Human Nibrin (NBN) and of Its 26 kDa and 70 kDa Fragments Arising from the *NBN* 657del5 Founder Mutation

**DOI:** 10.1371/journal.pone.0114651

**Published:** 2014-12-08

**Authors:** Domenica Cilli, Cristiana Mirasole, Rosa Pennisi, Valeria Pallotta, Angelo D'Alessandro, Antonio Antoccia, Lello Zolla, Paolo Ascenzi, Alessandra di Masi

**Affiliations:** 1 Department of Science, Roma Tre University, Rome, Italy; 2 Department of Ecological and Biological Sciences, University of Tuscia, Viterbo, Italy; 3 Istituto Nazionale Biostrutture e Biosistemi – Consorzio Interuniversitario, Rome, Italy; 4 Interdepartmental Laboratory for Electron Microscopy, Roma Tre University, Rome, Italy; St. Georges University of London, United Kingdom

## Abstract

Nibrin (also named NBN or NBS1) is a component of the MRE11/RAD50/NBN complex, which is involved in early steps of DNA double strand breaks sensing and repair. Mutations within the *NBN* gene are responsible for the Nijmegen breakage syndrome (NBS). The 90% of NBS patients are homozygous for the 657del5 mutation, which determines the synthesis of two truncated proteins of 26 kDa (p26) and 70 kDa (p70). Here, HEK293 cells have been exploited to transiently express either the full-length NBN protein or the p26 or p70 fragments, followed by affinity chromatography enrichment of the eluates. The application of an unsupervised proteomics approach, based upon SDS-PAGE separation and shotgun digestion of protein bands followed by MS/MS protein identification, indicates the occurrence of previously unreported protein interacting partners of the full-length NBN protein and the p26 fragment containing the FHA/BRCT1 domains, especially after cell irradiation. In particular, results obtained shed light on new possible roles of NBN and of the p26 fragment in ROS scavenging, in the DNA damage response, and in protein folding and degradation. In particular, here we show that p26 interacts with PARP1 after irradiation, and this interaction exerts an inhibitory effect on PARP1 activity as measured by NAD^+^ levels. Furthermore, the p26-PARP1 interaction seems to be responsible for the persistence of ROS, and in turn of DSBs, at 24 h from IR. Since some of the newly identified interactors of the p26 and p70 fragments have not been found to interact with the full-length NBN, these interactions may somehow contribute to the key biological phenomena underpinning NBS.

## Introduction

Human nibrin (also named NBN or NBS1), a component of the MRE11/RAD50/NBN (MRN) complex, has been reported to participate to cell cycle checkpoint activation, to the early steps of DNA damage sensing, and to double strand breaks (DSBs) repair [1]–[8]. The MRN complex accumulates at sites of DSBs in large microscopically discernible sub-nuclear structures, usually referred to as ionizing radiation (IR)-induced foci (IRIF) [9]. The foci formation around the DNA-damaged sites, marked by the presence of the H2AX histone phosphorylated at the Ser139 residue (*i.e.*, γ-H2AX), is mediated by a direct interaction between NBN and the phosphorylated mediator of the DNA damage checkpoint 1 (MDC1) [10]–[15]. Among others, NBN is known to interact also with ATM [10], [16]-[20], CtIP (also named RBBP8) [13], [21]–[27], Tip60 [28], [29], BRCA1 [23], [30], [31], and SMC1 [31].

**Figure 1:**
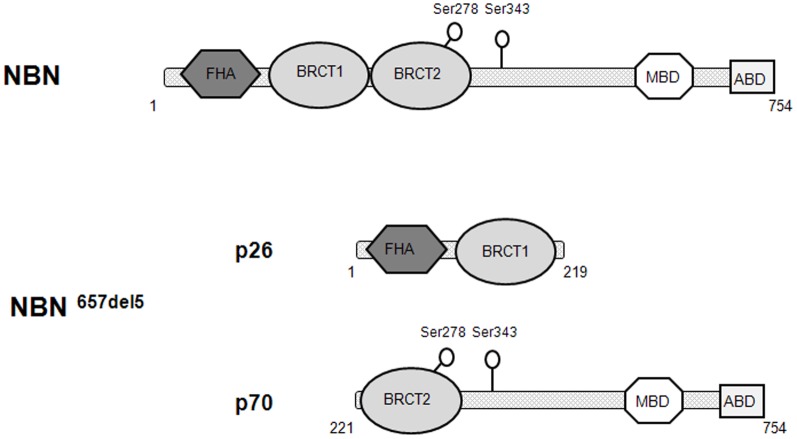
Schematic representation of the wild type and mutated NBN proteins. (**A**) Structure of the NBN wild type protein, having a molecular weight of approximately 85 kDa. (**B**) The 657del5 mutation, which splits up the tandem BRCT domains, determines the expression of two truncated proteins of 26 and 70kDa. The Ser278 (S278) and Ser343 (S343) residues are phosphorylated by ATM in response to the DNA damage induction. (FHA: fork-head associated domain; BRCT: breast cancer 1 (BRCA1) carboxy-terminal domain; MBD: MRE11 binding domain; ABM: ATM binding motif). For details, see the text.

NBN consists of 754 amino acids and is composed of three regions (Fig. 1). The *N*-terminus contains the fork-head associated (FHA) domain (amino acids 24-109) and two BRCA1 *C*-terminal (BRCT) tandem domains (*i.e.*, BRCT1: amino acids 114-183; BRCT2: amino acids 221-291) [32]. The central region of NBN contains two consensus sequences encompassing the Ser278 and Ser343 residues, which undergo phosphorylation by ATM in response to IR [33], [34]. The *C*-terminus of NBN contains two MRE11-binding motifs and the ATM-binding motif [13], [18], [19], [35].

Mutations at the homozygous or compound heterozygous status within the *NBN* gene are responsible for the Nijmegen breakage syndrome (NBS; OMIM #251260) [36], [37], a rare genetic disorder characterized by an autosomic recessive inheritance, whose signs are, among others, microcephaly, humoral and cellular immunodeficiency, and radiosensitivity [3], [6], [38]–[40]. Remarkably, one of the main clinical feature of NBS patients is the cancer predisposition. By the age of 20 years, over 40% of NBS patients develop a malignant disease, predominantly of lymphoid origin (Non-Hodgkin lymphomas of B and T cells are the most common) [40].

The 90% of NBS patients are homozygous for the hypomorphic 657del5 mutation in *NBN* [3], [40]. This mutation determines the synthesis of two truncated proteins of 26 kDa (p26) and 70 kDa (p70) (Fig. 1) [41]. The p26 protein includes the region encompassing amino acids 1-218 of the NBN protein, thus comprising the FHA and the BRCT1 domains. The p70 protein is produced by an alternative initiation of translation upstream the 5 base pair deletion; after a 18 residue extension at the *N*-terminus, the sequence is identical to that of the wild type NBN from the amino acid 221 to the end, and contains the BRCT2 domain and the *C*-terminal region of NBN [32]. Remarkably, a significant correlation between the p70 expression levels and lymphoma incidence has been observed; in fact, patients displaying high intracellular levels of the p70 truncated protein are at lower risk for lymphoma than those with low levels of p70 [42].

The aim of the present work was to identify, by SDS-PAGE and mass spectrometry approaches, the interactors of both the full-length NBN protein and of the p26 and p70 NBN fragments, under basal condition and after X-rays-induced DNA damage. Data obtained allowed the identification of several previously unreported interacting partners of NBN. A high number of interactors of the p26 (containing the FHA/BRCT1 domains) and p70 (containing the BRCT2 domain and the MRE11/ATM binding motifs) NBN fragments were isolated after DNA damage induction, most of them not interacting with the full-length NBN. Results obtained shed light on new possible roles of NBN in ROS scavenging, in the DNA damage response, as well as in protein folding and degradation.

## Materials and Methods

### Amplification and cloning

The p26 and p70 coding sequences were obtained from the total RNA of a lymphoblastoid cell line established from a NBS patient homozygous for the 657del5 mutation. Two micrograms of the RNA sample were reverse-transcribed using the SuperScript III First-Strand Synthesis System for RT-PCR (Invitrogen, Carlsbad, USA). The oligo-dT primer was used to prime the reverse transcription, which was performed according to the manufacturer's instructions. PCR was performed using the Exact Polymerase (5 Prime GmbH, Hamburg, Germany), using as templates the cDNA obtained from the reverse transcription. For the amplification of the NBN full-length sequence, the pLXIN-NBN retroviral vector was used as template [43]. The primer sequences, all carrying at the 5′ and 3′ ends the BsaI restriction enzyme recognition sites, were designed using the “Primer D'Signer Software” (IBA, Goettingen, Germany). Oligonucleotide sequences used were:

p26_FW: 5′ - ATGGTAGGTCTCAGCGCCATGTGGAAACTGCTGCCCGCC - 3′;

p26_RV: 5′ - ATGGTAGGTCTCATATCATGCTGTTTGGCATTCAAAAATATAAAT - 3′;

p70_FW: 5′ - ATGGTAGGTCTCAGCGCTTCTTCTCCTTTTTAAATAAGGATTGTA - 3′;

p70_RV: 5′ - ATGGTAGGTCTCAGCGCTTCTTCTCCTTTTTAAATAAGGATTGTA - 3′;

NBN_FW: 5′ - ATGGTAGGTCTCAGCGCCATGTGGAAACTGCTGCCCGCC - 3′;

NBN_RV:5′ - ATGGTAGGTCTCAGCGCTTCTTCTCCTTTTTAAATAAGGATTGTA - 3′.

The resulting amplicons were digested with the BsaI restriction enzyme (New England Biolabs, Ipswich, USA) and ligated into the BsaI pre-digested pEXPR-IBA105 vector (IBA) using the Quick Ligation kit (New England Biolabs). The resulting plasmids were sequenced to check the proper cDNA sequence insertion, amplified in the JM109 *E. coli* strain, and purified using the PerfectPrep Endofree Maxi kit (5 Prime).

### Cell culture and transient transfection

HEK293 cells were grown in Dulbecco Modified Eagle's medium supplemented with 10% fetal bovine serum (VWR, Milan, Italy), 2 mM L-glutamine (VWR), 100 µg/ml penicillin and 100 µg/ml streptomycin (VWR). Transient transfection was performed using the calcium chloride method [44]. Briefly, HEK293 cells were seeded at a density of 2×10^5^ cells/ml. One hour prior transfection the medium was refreshed and then 10 µg of plasmid DNA (either p26_pEXPR-IBA105 or p70_pEXPR-IBA105 or NBN_pEXPR-IBA105 or empty pEXPR-IBA105) was mixed together with 0.25 M CaCl_2_ in Hepes buffer (HBS: 25 mM Hepes, 10 mM KCl, 12 mM Dextrose, 280 mM NaCl, and 1.5 mM Na_2_HPO_4_×7H_2_O). To check the expression of the recombinant proteins fused to the Strep-tag sequence, HEK293 cells were lysed after 24 and 48 h from transfection (lysis buffer: 20 mM Tris-HCl pH 8.0, 137 mM NaCl, 10% glycerol (v/v), 1% NP-40 (v/v), 10 mM EDTA, 1 µg/mL aprotinin, 1 µg/mL leupeptin, 1 µg/mL pepstatin, 1.0 mM orthovanadate, and 2.0 mM PMSF). Thirty micrograms of protein lysates were analyzed by immunoblotting using a StrepMAB-Classic horse radish peroxidase conjugated antibody (IBA).

### X-ray treatment

In order to induce DSBs, cells were exposed to 2 Gy of X-rays 48 h after transfection (MGL 300/6-D apparatus, Gilardoni, Italy; 250 kV, 6 mA, Cu filter; dose rate 0.53 Gy/min).

### Strep-tag chromatography

Thirty minutes after X-ray treatment, untreated and irradiated cells were harvested and lysed using 400 µl (column bed volume, CV) of the lysis buffer. Columns packed with 5.0 ml of Strep-Tactin Sepharose resin (IBA) were equilibrated with 1 CV of buffer W. After centrifugation of soluble extracts (14,000 rpm, 5 minutes, 4°C), each supernatant was added to an equilibrated column. After the cell extracts have completely entered the columns, they were washed 5 times with 0.5 CV, and the eluates were collected in fractions having a size of 1 CV. Finally, 0.5 CVs of buffer E (100 mM TrisHCl, 150 mM NaCl, 1.0 mM EDTA pH 8.0, and 2.5 mM desthiobiotin pH 8.0) were added 6 times to each column and the eluates were collected in 0.5 CV fractions. Twenty µL samples of each fraction were analyzed by SDS-PAGE, using precast gradient polyacrylamide gels (BioRad, Hercules, USA). Proteins were detected by silver staining. Gel lanes were divided into 30 uniform slices and subjected to trypsin digestion for shotgun MS-based identification, as previously reported [45].

### MALDI TOF/TOF and nanoHPLC/Mass spectrometry

Protein slices were excised from the first dimension SDS-PAGE gels and subjected to trypsin digestion according to literature [46] with minor modifications. The gel pieces were swollen in a digestion buffer containing 50 mM NH_4_HCO_3_ and 12.5 ng/mL trypsin (modified porcine trypsin, sequencing grade, Promega, Madison, WI, USA) in an ice bath. After 30 min, the supernatant was removed and discarded; then 20 µL of 50 mM NH_4_HCO_3_ were added to the gel pieces and digestion was allowed to proceed overnight at 37°C. The supernatant containing tryptic peptides was dried by vacuum centrifugation prior to MALDI-TOF/TOF [47] and nano-liquid chromatography-electrospray ionization-ion trap mass spectrometry/mass spectrometry (nano-LC-ESI-IT MS/MS) identification [48].

MALDI-based identification was performed through an Autoflex II MALDI-TOF/TOF mass spectrometer. The LIFT module (Bruker Daltonics, Bremen, Germany) was used for mass analysis of peptide mixtures. Twenty microliters of the tryptic protein digests were loaded onto activated (0.1% TFA in acetonitrile) ZipTip columns and washed three times with 10 µL of 0.1% TFA in DD-H_2_O. The peptides were eluted with 1.0 µL of matrix solution (0.7 mg/mL α-cyano-4-hydroxy-trans-cinnamic acid (Fluka, Seelze, Germany) in 85% acetonitrile, 0.1% TFA and 1.0 mM NH_4_H_2_PO_4_) and spotted directly on the MALDI-TOF target plate for automatic identification (PAC384 pre-spotted anchor chip). Proteins were identified by peptide mass fingerprint (PMF) using the database search program MASCOT (http://www.matrixscience.com/) upon removal of background ion peaks. Accuracy was set within 50 ppm, while the enzyme chosen was trypsin and only 1 missed cleavage was allowed. Fixed carbamidomethyl Cys and variable Met-oxidation was used as optional search criterion. For those proteins for which PMF-based identification was not successful, most abundant peptides were analyzed with MALDI-TOF/TOF-based LIFT mode MS/MS analyses of precursor ions and repeated MASCOT-based database searches. Runs were performed automatically through FlexControl setting and Biotools processing of MS data. When PMF protein identification was unsuccessful, automatic determination of the three most abundant peaks and identification through MS/MS (LIFT analysis) on the three most intense ion peaks was performed. A peptide mixture (Peptide calibration standard I; Bruker Daltonics) was used for external calibration.

Nano-LC-ESI-IT MS/MS identification was performed on those protein bands or spots that could not be successfully identified either through PMF or LIFT (MS/MS) MALDI TOF/TOF analyses. Nano-LC-ESI-IT MS/MS analysis was performed through a split-free nano-flow chromatography separation system (EASY-nLC II, Proxeon, Odense, Denmark) coupled to a 3D-ion trap (model AmaZon ETD, Bruker Daltonik, Germany) equipped with an online ESI nano-sprayer (the spray capillary was a fused silica capillary, 0.020 mm i.d., 0.090 mm o.d.). For all experiments, a sample volume of 15 µL was loaded by the autosampler onto a homemade 2 cm fused silica pre-column (100 µm i.d., 375 µm o.d., Reprosil C18-AQ, 5 µm; Dr. Maisch GmbH, Ammerbuch-Entringen, Germany). Sequential elution of peptides was accomplished using a flow rate of 300 nL/min and a linear gradient from Solution A (2% acetonitrile; 0.1% formic acid) to 50% of Solution B (98% acetonitrile; 0.1% formic acid) in 40 min over the pre-column in-line with a homemade 15 cm resolving column (75 µm i.d., 375 µm o.d., Reprosil C18-AQ, 3 µm; Dr. Maisch GmbH, Ammerbuch-Entringen, Germany). The acquisition parameters were as follows: dry gas temperature, 220°C; dry gas, 4.0 L/min; nebulizer gas, 10 psi; electrospray voltage, 4000 V; high-voltage end-plate offset, −200 V; capillary exit, 140 V; trap drive: 63.2; funnel 1 in 100 V out 35 V, and funnel 2 in 12 V out 10 V; ICC target, 200,000 and maximum accumulation time, 50 ms. The sample was subjected to the “Enhanced Resolution Mode” at 8100 m/z per second (which allows mono isotopic resolution up to four charge stages) polarity positive, scan range from m/z 300 to 1500, 5 spectra averaged, and rolling average of 1. The “Smart Decomposition” was set to “auto”.

Acquired spectra were processed in DataAnalysis 4.0, and de-convoluted spectra were further analyzed with BioTools 3.2 software and submitted to the Mascot search program (in-house version 2.2, Matrix Science, London, UK). The following parameters were adopted for database searches: NCBInr database (release date 21/01/2012; 243,775 sequences); taxonomy: *Homo sapiens*; peptide and fragment mass tolerance: ±0.3 Da; enzyme specificity (trypsin) with 2 missed cleavages was considered; fixed modifications: carbamidomethyl and variable modifications: oxidation. For positive identification, the score of the result of (−10×Log P) had to be over the significance threshold level (p<0.05) and only peptides with Mascot scores ≧30 were considered. Peptide fragmentation spectra were also manually verified, accounting for the mass error, the presence of fragment ion series, and the expected prevalence of *C*-terminus containing (y-type ions) in the high mass range. All spots required a minimum of two verified peptides to be identified. Moreover, replicate measurements (*n* = 3) have confirmed the identity of these protein hits.

### Immunoblotting

To analyze the NBN full-length, the p26 and the p70 interactors identified by mass spectrometry, 30 µg of protein eluates obtained from the Strep-tag chromatography were loaded onto a polyacrylamide gel and transferred to a PVDF membrane (Immobilon, Millipore, Billerica, USA). The blots were probed with the following antibodies: anti-53BP1 rabbit polyclonal antibody (Novus Biologicals, Atlanta, USA), anti-ATM mouse monoclonal antibody (Abcam, Cambridge, UK), anti-ATM pSer1981 mouse monoclonal antibody (Santa Cruz Biotechnology, Dallas, USA), anti-BRCA1 mouse monoclonal antibody (Santa Cruz Biotechnology), anti-CHK2 pThr68 rabbit polyclonal antibody (Santa Cruz Biotechnology), anti-CHK2 mouse monoclonal antibody (Santa Cruz Biotechnology), anti-CtIP rabbit polyclonal antibody (Abcam), γ-H2AX rabbit polyclonal antibody (Santa Cruz Biotechnology), anti-Hsp90 rabbit polyclonal antibody (Santa Cruz Biotechnology), anti-MRE11 mouse monoclonal antibody (Santa Cruz Biotechnology), anti-NBN mouse monoclonal antibody (Abcam), anti-PP2A rabbit polyclonal antibody (Santa Cruz Biotechnology), anti-PARP1 mouse monoclonal antibody (Santa Cruz Biotechnology), anti-PML rabbit polyclonal antibody (Santa Cruz Biotechnology), anti-SMC1 rabbit polyclonal antibody (Chemicon, Billerica, USA), anti-Strep-tag (IBA). ECL detection solutions (Amersham Pharmacia, Milan, Italy) were used to visualize the antibody reactions.

### Protein-protein interaction analysis

The STRING 9.1 software [49] has been used to perform functional annotations, through mapping protein interactors, to the experimentally identified protein species. Proteins were uploaded in the software along with indications of the species under investigation (*Homo sapiens*) in order to exclude false-positive protein-protein interactions and functional annotations derived from investigations on other species. Protein-protein interaction analysis/prediction software, such as String, determines and makes graphs of unbiased networks, in which gene products are represented as nodes, and the biological relationship between two nodes is represented as an edge (line). All edges are supported by at least one reference from the literature, from a textbook, or from canonical information stored in the software internal database (literature and evidence-based). White nodes represent predicted interactors upon matching against the internal database which were not present in the uploaded list (absent in the experimental dataset). The confidence interval was set to 0.400 (high confidence), additional white nodes to 10, and network depth was kept to the minimum value 1 to exclude as many false positive interactions as possible.

### Quantification of NAD^+^ levels

Cells transfected with either the NBN full-length- or the p26-coding vector were treated with 2 Gy of X-rays and harvested after 0.5, 2, and 24 h. Briefly, 2×10^5^ cells were washed in PBS and lysed. To remove NADH-consuming enzymes, the extracted samples were filtered through 10 kDa molecular weight cut off filters (Abcam) before performing the assay. The NAD^+^/NADH quantification was performed using the NAD^+^/NADH colorimetric kit, according to manufacturer's instruction (BioVision, Milpitas, USA). Samples were read at OD 450 nm, using the microplate reader Victor X (PerkinElmer, Waltham, USA).

### Immunofluorescence analysis of γ-H2AX foci

HEK293 transfected cells were grown on glass coverslips, irradiated with 2 Gy of X-rays, and harvested after 0.5, 2, and 24 h. Cells were fixed in 2% paraformaldehyde, permeabilized on ice for 5 min with 0.2% Triton X-100, and blocked in PBS/1% BSA (v/w) for 0.5 h at room temperature. Slides were incubated over night with 1 µg/mL γ-H2AX mouse monoclonal antibody (Millipore, Billerica, MA), and detected with an anti-mouse FITC-conjugated secondary antibody (Immunological Sciences, Rome, Italy). Confocal analysis was performed using LCS microscope (Leica Microsystems, Heidelberg, Germany). Quantitative analysis of γ-H2AX foci was carried out by counting foci in at least 50 cells/experiment, in two repeated experiments.

### Statistical analysis

The statistical analysis was performed using the ANOVA test with the InStat version 3 software system (GraphPad Software Inc., San Diego, CA). Data are means of at least two independent experiments ± SD.

## Results and Discussion

### Recombinant proteins expression

**Figure 2:**
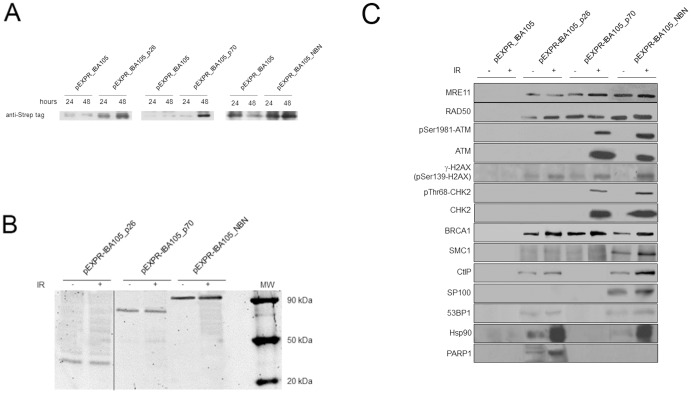
Evaluation of the Strep-tag recombinant proteins expression and Western blot analysis of NBN, p26 and p70 interactors. (**A**) Protein lysates, obtained after 24 and 48 h from HEK293 transient tranfections, were analyzed by Western blot using a StrepMAB-Classic horse radish peroxidase conjugated antibody. The highest level of expression was observed after 48 h from transfection, for all the recombinant proteins. Protein extracts obtained from HEK293 cells transfected with the empty vector were used as negative control. (**B**) Check of the expression of the Strep-tag recombinant proteins. Immunoblots were performed using the protein eluates obtained from HEK293 transfected cells either untreated or exposed to 2 Gy of X-rays, lysed after 30 minutes and purified by Strep-tag chromatography. Filters were probed with the anti-NBN antibody directed against the full-length protein. (**C**) Protein eluates obtained from HEK293 transfected cells exposed to 2 Gy of X-rays were lysed after 30 minutes, purified by Strep-tag chromatography, and analysed by Western blot using the following antibodies: anti-53BP1 rabbit polyclonal, anti-ATM mouse monoclonal, anti-ATM pSer1981 mouse monoclonal, anti-BRCA1 mouse monoclonal, anti-CHK2 pThr68 rabbit polyclonal, anti-CHK2 mouse monoclonal, anti-CtIP rabbit polyclonal, γ-H2AX rabbit polyclonal, anti-Hsp90 rabbit polyclonal, anti-MRE11 mouse monoclonal, anti-NBN mouse monoclonal, anti-PARP1 mouse monoclonal, anti-PP2A rabbit polyclonal, anti-PML rabbit polyclonal, anti-SMC1 rabbit polyclonal.

To identify the NBN full-length and the p26 and the p70 interactors, the cDNAs were cloned into the pEXPR-IBA105 plasmid, in frame with the Strep-tag coding sequence. The obtained plasmids, named pEXPR-IBA105_NBN, pEXPR-IBA105_p26, and pEXPR-IBA105_p70, respectively, were transiently transfected into HEK293 cells. The expression of the recombinant proteins fused to the Strep-tag was checked after 24 and 48 h from transfection, using the StrepMAB-Classic horse radish peroxidase conjugated antibody (IBA). After 48 h from transfection, the recombinant proteins were expressed at higher levels with respect to HEK293 cell transfected with the empty vector (named pEXPR-IBA105) (Fig. 2A).

### Identification of the interactors of NBN and p26 and p70 fragments arising from the *NBN* 657del5 founder mutation

HEK293 transfected cells were then exposed to 2 Gy of X-rays, and soluble cell extracts were prepared after 30 minutes. The NBN full-length, the p26, and the p70 recombinant proteins, together with the bound proteins present in the cell extracts, were purified using the Strep-tactin gravity flow columns. The obtained eluates were analyzed by mass spectrometry and SDS-PAGE.

Protein bands were excised from the gels and identified either through MALDI TOF/TOF or nanoHPLC-ESI MS/MS. In Tables 1-4 are reported the interactors identified for NBN, p26, p70, and the empty vector, respectively, in both untreated (K) and irradiated (RX) samples, along with the theoretical molecular weight and pI, the overall number of peptides identified, the Mascot score, and the explanation of the biological functions, as gleaned via the UniProt database. Table 5 summarizes the identified interactors of NBN full-length, and of p26 and p70 fragments, upon exclusion of false positive (hits highlighted in yellow in Table 1-4). This workflow allowed us to determine novel likely interactors of the full-length NBN protein, and of the p26 and p70 fragments, while expanding or complementing existing literature [15], [23], [25], [31], [50]-[53].

**Table 1:** NBN protein interactors.

	Mr, Da	pI	N° of peptides identified	Mascot Score	NCBI Accession Number	Protein ID [Homo sapiens]	Abbr.	Biological process
**K**	70267	5.48	31	1295	gi|4529892	**HSP70-2**	**HSPA1A**	**Folding of newly translated proteins in the cytosol and in organelles; ubiquitin-proteasome pathway; G2/M transition of mitotic cell cycle; meiosis**
	40194	5.55	16	546	gi|15277503	**Beta actin**	**ACTB**	**Constitution of the contractile apparatus**
	42480	5.23	9	359	gi|178027	**Alpha actin**	**ACTA1**	**Cell growth; cytoskeleton structural constitution; protein binding**
	50451	9.10	9	346	gi|4503471	**Elongation factor 1-alpha 1**	**EEF1A1**	**Proteins biosynthesis**
	61187	5.70	9	340	gi|31542947	**60 kDa heat shock protein, mitochondrial**	**HSPD1**	**Signaling in the innate immune system; folding and assembly of newly imported proteins in the mitochondria**
	47421	7.01	7	324	gi|693933	**2-phosphopyruvate-hydratase alpha enolase**	**ENO1**	**Phosphopyruvate hydratase activity; regulation of cell growth and transcription; tumor suppression**
	36202	8.26	6	282	gi|31645	**Glyceraldehyde-3-phosphate dehydrogenase**	**GAPDH**	**Glyceraldehyde-3-phosphate dehydrogenase activity; microtubule cytoskeleton organization; protein stabilization; translation regulation**
	83584	4.97	6	239	gi|306891	**90kDa heat shock protein**	**HSP90AB1**	**Unfolded protein binding; innate immune response; protein import into nucleus positive regulation; positive regulation of cell size**
	62570	7.01	4	147	gi|73535278	**Pyruvate Kinase**	**PKM2**	**Pyruvate kinase activity in glycolysis; protein-protein interactions; nuclear transport; programmed cell death**
	42745	7.01	2	121	gi|180555	**Creatine kinase-B**	**CKB**	**Creatine kinase activity; ATP and protein binding; brain development**
	75694	8.43	2	112	gi|1082886	**Tumor necrosis factor type 1 receptor associated protein TRAP-1-human**	**TRAP1**	**ATPase activity; cellular stress responses regulation**
	96246	6.41	4	111	gi|4503483	**Elongation factor 2**	**EEF2**	**Protein biosynthesis**
	36630	7.08	2	105	gi|4504973	**L-lactate dehydrogenase C chain**	**LDHC**	**Anaerobic glycolysis; possible role in sperm motility**
**RX**	70267	5.48	23	971	gi|4529892	**HSP70-2**	**HSPA1A**	**Folding of newly translated proteins in the cytosol and in organelles; ubiquitin-proteasome pathway; G2/M transition of mitotic cell cycle; meiosis**
	42933	5.34	6	290	gi|49457530	**Creatine kinase B**	**CKB**	**Creatine kinase activity; ATP and protein binding; brain development**
	37707	4.87	6	263	gi|18204869	**TUBA1B protein**	**TUBA1B**	**Cytoskeleton constitution; protein folding; cell division**
	40194	5.55	8	252	gi|15277503	**Beta actin**	**ACTB**	**Cytoskeleton constitution; protein folding**
	47421	7.01	6	223	gi|693933	**2-phosphopyruvate-hydratase alpha enolase**	**ENO1**	**Phosphopyruvate hydratase activity; regulation of cell growth and transcription; tumor suppression**
	71082	5.37	6	213	gi|5729877	**Heat shock cognate 71 kDa protein isoform 1**	**HSPA8**	**Proteins correct folding facilitation; transport of membrane components through the cell**
	50451	9.10	6	159	gi|4503471	**Elongation factor 1-alpha 1**	**EEF1A1**	**Proteins biosynthesis**
	61187	5.70	4	154	gi|31542947	**60 kDa heat shock protein, mitochondrial**	**HSPD1**	**Signaling in the innate immune system; folding and assembly of newly imported proteins in the mitochondria**
	36202	8.26	3	135	gi|31645	**Glyceraldehyde-3-phosphate dehydrogenase**	**GAPDH**	**Glyceraldehyde-3-phosphate dehydrogenase activity; microtubule cytoskeleton organization; protein stabilization; translation regulation**
	63839	5.03	3	125	gi|3287489	**Hsp89-alpha-delta-N**	**HSP90AA1**	**Response to unfolded protein; ubiquitin-proteasome pathway; G2/M progression; meiosis**
	46353	5.32	2	111	gi|4503529	**Eukaryotic initiation factor 4AII**	**EIF4A2**	**ATP binding; helicase activity; protein binding; translational initiation**
	39706	8.34	3	102	gi|28614	**Aldolase A**	**ALDOA**	**Fructose-bisphosphate aldolase activity; cytoskeletal protein binding; regulation of cell shape; striated muscle contraction; actin filament organization**
	29195	8.79	2	100	gi|187074	**L-lactate dehydrogenase C chain**	**LDHC**	**Anaerobic glycolysis; possible role in sperm motility**
	75694	8.43	2	92	gi|1082886	**Tumor necrosis factor type 1 receptor associated protein TRAP-1 -human**	**TRAP1**	**ATPase activity; cellular stress responses regulation**
	56525	5.26	2	90	gi|32189394	**ATP synthase subunit beta, mitochondrial precursor**	**ATP5B**	**ATP synthesis**
	3737	6.86	2	79	gi|913148	**Calreticulin = calcium binding protein**	**CALR**	**Ca(2+)-dependent proteins folding; oligomeric assembly and quality control in the endoplasmic reticulum (ER)**
	16096	5.86	2	79	gi|1237406	**Cu/Zn-superoxide dismutase**	**SOD1**	**Superoxide radicals to molecular oxygen and hydrogen peroxide conversion**
	19135	6.41	3	75	gi|55959887	**Peroxiredoxin 1**	**PRDX1**	**Thioredoxin peroxidase activity; cell proliferation; skeletal system development**
	58411	7.58	2	52	gi|35505	**Pyruvate Kinase**	**PKM2**	**Pyruvate kinase activity in glycolysis; protein-protein interactions; nuclear transport; programmed cell death**
	42480	5.23	6	197	gi|178027	**Alpha actin**	**ACTA1**	**Cell growth; cytoskeleton structural constitution; protein binding**
	31888	4.84	2	114	gi|34234	**Laminin-binding protein**	**RPSA**	**Assembly and/or stability of the 40S ribosomal subunit; cell adhesion to the basement membrane and consequent activation of signaling transduction pathways; cell fate determination; tissue morphogenesis**

**Table 2:** p26 fragment interactors.

	Mr, Da	pI	N° of peptides identifid	Mascot Score	NCBI Accession Number	Protein ID [Homo sapiens]	Abbr.	Biological process
**K**	95565	5.85	11	74	gi|119618310	**ATPase, Ca^2+^ transporting, cardiac muscle, slow twitch 2, isoform CRA_c**	**ATP2A2**	**Catalysis of the hydrolysis of ATP coupled with the translocation of calcium from the cytosol to the sarcoplasmic reticulum lumen; implication of isoform 2 in the regulation of the contraction/relaxation cycle**
	52576	6.15	1 MS/MS	37	gi|12655059	**G protein-coupled receptor kinase interacting ArfGAP 2**	**GIT2**	**Interaction with G protein-coupled receptor kinases; association with paxillin**
	33149	6.71	6	73	gi|197210450	**Uridine monophosphate synthetase isoform H**	**UMPS**	**Pyrimidine biosynthesis**
	225582	6.09	13	71	gi|119590850	**Phosphatidylinositol-3-phosphate/phosphatidylinositol 5-kinase, type III, isoform CRA_a**	**PIKFYVE**	**Catalysis of the reaction: a 1-phosphatidyl-1D-myo-inositol 3-phosphate + ATP = a 1-phosphatidyl-1D-myo-inositol 3,5-bisphosphate + ADP + 2 H(+)**
	18818	8.64	6	67	gi|169145006	**Proline rich 5**	**PRR5**	**Regulation of cell growth and survival in response to hormonal signals; regulation of the actin cytoskeleton by mTORC2; action as a tumor suppressor in breast cancer**
	29195	8.79	4	67	gi|187074	**L-lactate dehydrogenase C chain**	**LDHC**	**Anaerobic glycolysis; possible role in sperm motility**
	111302	6.80	7	72	gi|558305	**Dihydropyrimidine dehydrogenase**	**DPYD**	**Pyrimidine base degradation; degradation of the chemotherapeutic drug 5-fluorouracil**
	29031	8.72	7	68	gi|197102783	**Carbonic anhydrase-related protein 10**	**CA10**	**No catalytic activity.**
	90404	6.83	11	78	gi|119629553	**Rho guanine nucleotide exchange factor (GEF) 7, isoform CRA_f**	**ARHGEF7**	**Cell migration, attachment and cell spreading; possible function as a positive regulator of apoptosis; spines and synapses formation promotion in hippocampal neurons**
	4999	8.94	4	70	gi|5726470	**Fc gamma receptor III-A**	**FCGR3A**	**Binding of complexed or aggregated IgG and also monomeric IgG; mediation of antibody-dependent cellular cytotoxicity (ADCC) and other antibody-dependent responses, such as phagocytosis**
	24735	7.86	7	77	gi|119582864	**Phosphoglucomutase 5**	**PGM5**	**Adherens-type cell-cell and cell-matrix junctions formation**
**RX**	40194	5.55	5	454	gi|15277503	**Beta actin**	**ACTB**	**Cytoskeleton structural constitution; axon guidance; cellular component movement**
	14083	10.90	1	125	gi|4504239	**Histone H2A type 1**	**HIST1H2AK**	**Nucleosome assembly; DNA binding**
	473749	6.75	27	496	Ngi|13654237 cession	**DNA-dependent protein kinase catalytic subunit isoform 1**	**PRKDC**	**Double-strand break repair via nonhomologous end joining; response to gamma radiation; immunoglobulin V(D)J recombination; telomere maintenance; germ cell programmed cell death; DNA-dependent protein kinase activity; apoptotic process**
	275542	5.98	9	147	gi|915392	**Fatty acid synthase**	**FASN**	**Fatty acid synthase activity; drug binding; protein homodimerization activity**
	245100	6.00	2	114	gi|1228049	**Multifunctional protein CAD**	**CAD**	**Carbamoyl-phosphate synthase (glutamine-hydrolyzing) activity; cellular response to drug; organ regeneration; embryo development**
	193260	5.48	64	1397	gi|4758012	**Clathrin heavy chain 1**	**CLTC**	**Mitosis; axon guidance; intracellular protein transport**
	145700	5.82	5	226	gi|440799	**Isoleucyl-tRNA synthetase**	**IARS**	**tRNA aminoacylation for protein translation; ATP binding; gene expression**
	115243	5.87	1	175	gi|119611543	DEAH (Asp-Glu-Ala-His) box polypeptide 9, isoform CRA_a*	**DHX9**	**DNA and protein binding; double-stranded RNA binding; RNA helicase activity; RNA splicing; cellular response to heat**
	146464	6.58	2	82	gi|4033735	**Spliceosomal protein SAP 155**	**SF3B1**	**Chromatin binding; RNA splicing; gene expression**
	89631	5.60	26	468	gi|32358	**HnRNP U protein**	**HNRNPU**	**DNA binding; RNA splicing**
	113810	9.02	9	186	gi|190167	**Poly(ADP-ribose) polymerase**	**PARP1**	**DNA damage response, detection of DNA damage; base-excision repair; regulation of growth rate; telomere maintenance**
	11360	11.36	3	225	gi|4504301	**Histone H4**	**HIST1H4A**	**DNA binding; telomere maintenance; phosphatidylinositol-mediated signaling**
	122882	5.83	3	209	gi|134133226	**POTE ankyrin domain family member E**	**POTEE**	**ATP binding**
	42318	5.39	3	205	gi|63055057	**Beta-actin-like protein 2**	**ACTBL2**	**ATP binding**
	47421	7.01	4	158	gi|693933	**2-phosphopyruvate-hydratase alpha enolase**	**ENO1**	**Phosphopyruvate hydratase activity; regulation of cell growth and transcription; tumor suppression**
	53738	5.03	4	128	gi|340219	**Vimentin**	**VIM**	**Protein binding; cytoskeleton structural constitution; apoptotic process; cellular component movement**
	83242	4.97	1 MS/MS	68	gi|306891	**90kDa heat shock protein**	**HSP90AB1**	**Unfolded protein binding; innate immune response; protein import into nucleus positive regulation; positive regulation of cell size**
	72402	5.07	23	434	gi|16507237	**78 kDa glucose-regulated protein precursor**	**HSPA5**	**Misfolded and unfolded protein binding; negative regulation of apoptotic process**
	69825	5.42	12	105	gi|386785	**Hsp89-alpha-delta-N**	**HSP90AA1**	**Response to unfolded protein; ubiquitin-proteasome pathway; G2/M progression; meiosis**
	50126	5.02	1 MS/MS	123	gi|37492	**Alpha tubulin**	**TUBA1A**	**Cytoskeleton structural constitution; G2/M transition of mitotic cell cycle; cell division; protein folding and proliferation**
	33149	6.71	6	71	gi|197210450	**Uridine monophosphate synthetase isoform H**	**UMPS**	**Pyrimidine biosynthesis**
	24475	10.32	4	329	gi|4506743	**40S ribosomal protein S8**	**RPS8**	**Ribosome structural constitution; translation**
	29326	4.63	4	235	gi|5803225	**14-3-3 protein epsilon**	**YWHAE**	**Phosphoprotein binding; protein complex binding; G2/M transition of mitotic cell cycle; apoptotic process; hippocampus development**
	35542	6.97	4	225	gi|30354619	**YWHAZ protein, partial**	**YWHAZ**	**Protein binding; apoptotic process; signal transduction**
	28057	8.60	2	206	gi|4092058	**Proteasome subunit HSPC**	**PSMA7**	**DNA damage response, signal transduction by p53 class mediator resulting in cell cycle arrest; G1/S transition of mitotic cell cycle; apoptotic process; cell cycle checkpoint; protein polyubiquitination**
	23610	5.21	4	167	gi|542991	**Ran-specific GTPase-activating protein - human**	**RANBP1**	**GTPase activator activity; intracellular transport; intracellular transport**
	26894	7.10	6	478	gi|136066	**RecName: Full = Triosephosphate isomerase; Short = TIM; AltName: Full = Triose-phosphate isomerase**	**TPI1**	**Triose-phosphate isomerase activity; embryo development**
	24806	6.70	7	450	gi|14250587	**L-isoaspartate (D-aspartate) O-methyltransferase protein**	**PCMT1**	**Protein repair; protein-L-isoaspartate (D-aspartate) O-methyltransferase activity**
	23565	11.5	2	226	gi|4506609	**60S ribosomal protein L19**	**RPL19**	**Ribosome structural constitution; RNA binding; translation**
	23506	5.43	2	178	gi|5822569	**Chain A, Crystal Structure Of HGSTP1-1[V104] complexed with the GSH conjugate Of (+)-Anti-BPDE**	**GSTP1**	**Conjugation of reduced glutathione to a wide number of exogenous and endogenous hydrophobic electrophiles; negative regulation of CDK5 activity via p25/p35 translocation to prevent neurodegeneration**
	24519	7.01	3	69	gi|5107637	**Chain C, Structure Of The Karyopherin Beta2-Ran Gppnhp Nuclear Transport Complex**	**TNPO1**	**Cell cycle; protein transport; gene expression**
	22324	8.27	4	221	gi|4505591	**Peroxiredoxin-1**	**PRDX1**	**Thioredoxin peroxidase activity; cell proliferation; skeletal system development**
	25527	4.80	2	165	gi|558528	**Proteasome subunit Y**	**PSMB6**	**DNA damage response, signal transduction by p53 class mediator resulting in cell cycle arrest; G1/S transition of mitotic cell cycle; apoptotic process; cell cycle checkpoint; protein polyubiquitination**
	17882	11.26	2	227	gi|4506619	**60S ribosomal protein L24**	**RPL24**	**Ribosome structural constitution; RNA binding; translation**
	22635	10.66	2	89	gi|14141193	**40S ribosomal protein S9**	**RPS9**	**Ribosome structural constitution; translation; positive regulation of cell proliferation**
	14835	9.21	3	549	gi|4506613	**60S ribosomal protein L22 proprotein**	**RPL22**	**Ribosome structural constitution; RNA binding; translation**
	18098	7.82	4	222	gi|1633054	**Cyclophilin A Complexed With Dipeptide Gly-Pro, Chain A**	**PPIA**	**PPIases associated with the proteins folding acceleration**
	16434	10.07	3	190	gi|5032051	**40S ribosomal protein S14**	**RPS14**	**Ribosome structural constitution; RNA binding; translation; ribosomal small subunit assembly**
	58411	7.58	2	294	gi|35505	**Pyruvate kinase**	**PKLR**	**Pyruvate kinase activity in glycolysis; protein-protein interactions; nuclear transport; programmed cell death**

**Table 3:** p70 fragment interactors.

	Mr, Da	pI	N° of peptides identified	Mascot Score	NCBI Accession Number	Protein ID [Homo sapiens]	Abbr.	Biological process
**K**	50804	4.94	2	137	gi|340021	**Alpha-tubulin**	**TUBA1A**	**Cytoskeleton structural constitution; G2/M transition of mitotic cell cycle; cell division; protein folding and proliferation**
	70110	5.42	16	607	gi|386785	**Heat shock protein**	**HSF1**	**Response to unfolded protein; ubiquitin-proteasome pathway; G2/M progression; meiosis**
	42128	5.22	11	303	gi|15277503	**Beta actin**	**ACTB**	**Cytoskeleton structural constitution; protein folding; cellular component movement**
	9543	5.80	3	173	gi|3153859	**Thioredoxin delta 3**	**TXN**	**Response to radiation; cell proliferation; innate immune response; oxidation-reduction process**
	29326	4.63	3	170	gi|5803225	**14-3-3 protein epsilon**	**YWHAE**	**Phosphoprotein binding; protein complex binding; G2/M transition of mitotic cell cycle; apoptotic process; hippocampus development**
	24919	4.50	2	132	gi|4503477	**Elongation factor 1-beta**	**EEF1B2**	**Translation elongation factor activity; protein binding**
	39706	8.34	2	83	gi|28614	**Aldolase A**	**ALDOA**	**Fructose-bisphosphate aldolase activity; cytoskeletal protein binding; regulation of cell shape; striated muscle contraction; actin filament organization**
**RX**	42745	5.34	11	295	gi|180555	**Creatine kinase-B**	**CKB**	**Creatine kinase activity; ATP and protein binding; brain development**
	46593	5.33	2	92	gi|485388	**Eukaryotic initiation factor 4AII**	**EIF4A2**	**ATP binding; helicase activity; protein binding; translational initiation**
	70267	5.48	14	553	gi|4529892	**HSP70-2**	**HSPA1A**	**Folding of newly translated proteins in the cytosol and in organelles; ubiquitin-proteasome pathway; G2/M transition of mitotic cell cycle; meiosis**
	42128	5.48	11	5.22	gi|28336	**Mutant beta-actin (beta'-actin)**	**ACTB**	**Cytoskeleton structural constitution; protein folding; cellular component movement**
	29326	4.63	7	248	gi|5803225	**14-3-3 protein epsilon**	**YWHAE**	**Phosphoprotein binding; protein complex binding; G2/M transition of mitotic cell cycle; apoptotic process; hippocampus development**
	42366	5.23	7	221	gi|4501881	**Alpha actin**	**ACTA1**	**Cell growth; cytoskeleton structural constitution; protein binding**
	37707	4.87	4	219	gi|18204869	**TUBA1B protein**	**TUBA1B**	**Cytoskeleton constitution; protein folding; cell division**
	83584	4.97	5	205	gi|306891	**90kDa heat shock protein**	**HSP90AB1**	**Unfolded protein binding; innate immune response; protein import into nucleus positive regulation; positive regulation of cell size**
	24919	4.50	2	155	gi|4503477	**Elongation factor 1-beta**	**EEF1B2**	**Translation elongation factor activity; protein binding**
	39706	8.34	3	150	gi|28614	**Aldolase A**	**ALDOA**	**Fructose-bisphosphate aldolase activity; cytoskeletal protein binding; regulation of cell shape; striated muscle contraction; actin filament organization**
	42318	5.39	3	122	gi|63055057	**Beta-actin-like protein 2**	**ACTBL2**	**ATP binding**

**Table 4:** Empty vector (false positive).

Mr, Da	pI	N° of peptides identified	Mascot Score	NCBI Accession Number	Protein ID [Homo sapiens]	Abbr.	Biological Process
36031	8.26	1 MS/MS	76	gi|31645	**Glyceraldehyde-3-phosphate dehydrogenase**	**GAPDH**	**Glyceraldehyde-3-phosphate dehydrogenase activity; microtubule cytoskeleton organization; protein stabilization; translation regulation**
275542	5.98	7	149	gi|915392	**Fatty acid synthase**	**FASN**	**Fatty acid synthase activity; drug binding; protein homodimerization activity**
83242	4.97	1 MS/MS	98	gi|306891	**90kDa heat shock protein**	**HSP90B1**	**Unfolded protein binding; innate immune response; protein import into nucleus positive regulation; positive regulation of cell size**
72402	5.07	13	244	gi|16507237	**78 kDa glucose-regulated protein precursor**	**HSPA5**	**Misfolded and unfolded protein binding; negative regulation of apoptotic process**
70009	5.48	13	148	gi|4529892	**HSP70-2**	**HSPA1A**	**Unfolded protein binding; protein refolding; cell proliferation and growth negative regulation; negative regulation of apoptotic process**
50126	5.02	1 MS/MS	115	gi|37492	**Alpha-tubulin**	**TUBA1A**	**Cytoskeleton structural constitution; G2/M transition of mitotic cell cycle; cell division; protein folding and proliferation**
40194	5.55	9	124	gi|15277503	**Beta actin**	**ACTB**	**Cytoskeleton structural constitution; protein folding; cellular component movement**
118938	9.57	7	77	gi|4566495	**Topoisomerase I-binding RS protein**	**TOPORS**	**DNA binding; intrinsic apoptotic signaling pathway in response to DNA damage; SUMO ligase activity; regulation of cell proliferation; transcription, DNA-dependent**
29326	4.63	14	338	gi|5803225	**14-3-3 protein epsilon**	**YWHAE**	**Phosphoprotein binding; protein complex binding; G2/M transition of mitotic cell cycle; apoptotic process; hippocampus development**
4999	8.94	4	70	gi|5726470	**Fc gamma receptor III-A**	**FCGR3A**	**Binding of complexed or aggregated IgG and also monomeric IgG; mediation of antibody-dependent cellular cytotoxicity (ADCC) and other antibody-dependent responses, such as phagocytosis**
24735	7.86	7	77	gi|119582864	**Phosphoglucomutase 5**	**PGM5**	**Adherens-type cell-cell and cell-matrix junctions formation**

**Table 5:** Novel interactors of NBN, p26 and p70 fragments.

	Abbr.	NBN		p26		p70	
Irradiation		−	+	−	+	−	+
2-phosphopyruvate-hydratase alpha enolase	ENO1	x	x		x		
40S ribosomal protein S14	RPS14				x		
40S ribosomal protein S8	RPS8				x		
40S ribosomal protein S9	RPS9				x		
60S ribosomal protein L19	RPL19				x		
60S ribosomal protein L22	RPL22				x		
60S ribosomal protein L24	RPL24				x		
Aldolase	ALDOA		x			x	x
Alpha actin	ACTA1	x	x				x
ATP synthase subunit beta, mitochondrial precursor	ATP5B		x				
ATPase, Ca^2+^ transporting, cardiac muscle, slow twitch 2, isoform CRA_c	ATP2A2			x			
Beta-actin like protein 2	ACTBL2				x		x
Calreticulin = calcium binding protein	CALR		x				
Carbonic anhydrase-related protein 10	CA10			x			
Chain C, Structure Of The Karyopherin Beta2-Ran Gppnhp Nuclear Transport Complex	TNPO1				x		
Clathrin heavy chain 1	CLTC				x		
Creatine kinase B	CKB	x	x				x
Cu/Zn-superoxide dismutase	SOD1		x				
Cyclophilin A Complexed With Dipeptide Gly-Pro, Chain A	PPIA				x		
DEAH (Asp-Glu-Ala-His) box polypeptide 9, isoform CRA_a*	DHX9				x		
Dihydropyrimidine dehydrogenase	DPYD			x			
DNA-dependent protein kinase catalytic subunit isoform 1	PRKDC				x		
Elongation factor 1 alpha 1	EEF1A1	x	x				
Elongation factor 1 beta	EEF1B2					x	x
Elongation factor 2	EEF2	x					
Eukaryotic initiation factor 4A	EIF4A2		x				x
G protein-coupled receptor kinase interacting ArfGAP 2	GIT2			x			
Heat shock cognate 71 kDa protein isoform 1	HSPA8		x				
Histone H2A type 1	HIST1H2AK				x		
Histone H4	HIST1H4A				x		
HnRNP U	HNRNPU				x		
Hsp89-alpha-delta-N	HSP90AA1		x		x		
Isoleucyl-tRNA synthetase	IARS				x		
Laminin-binding protein	RPSA		x				
L-isoaspartate (D-aspartate) O-methyltransferase protein	PCMT1				x		
L-lactate dehydrogenase C chain	LDHC	x	x	x			
Multifunctional protein CAD	CAD				x		
Peroxiredoxin 1	PRDX1		x		x		
Phosphatidylinositol-3-phosphate/phosphatidylinositol 5-kinase, type III, isoform CRA_a	PIKFYVE			x			
Poly(ADP)-ribose polymerase	PARP1				x		
POTE ankyrin domain family member E	POTEE				x		
Proline rich 5	PRR5			x			
Proteasome subunit HSPC	PSMA7				x		
Proteasome subunit Y	PSMB6				x		
Pyruvate kinase	PKM2/PKLR	x	x		x		
Ran-specific GTPase-activating protein - human	RANBP1				x		
RecName: Full = Triosephosphate isomerase; Short = TIM; AltName: Full = Triose-phosphate isomerase	TPI1				x		
Rho guanine nucleotide exchange factor (GEF) 7, isoform CRA_f	ARHGEF7			x			
Spliceosomal protein SAP 155	SF3B1				x		
Thioredoxin delta 3	TXN					x	
Tumor necrosis factor type 1 receptor associated protein TRAP-1 -human	TRAP1	x	x				
Uridine monophosphate synthetase isoform H	UMPS			x	x		
Vimentin	VIM				x		
YWHAZ protein, partial	YWHAZ				x		

The protein eluates obtained from the Strep-tag chromatography performed on lysates of HEK293 transfected with either NBN full-length, p26 or p70 were checked for the presence of the recombinant proteins, using an antibody directed against the full-length protein (Fig. 2B).

#### Interactors of the full-length NBN

**Figure 3:**
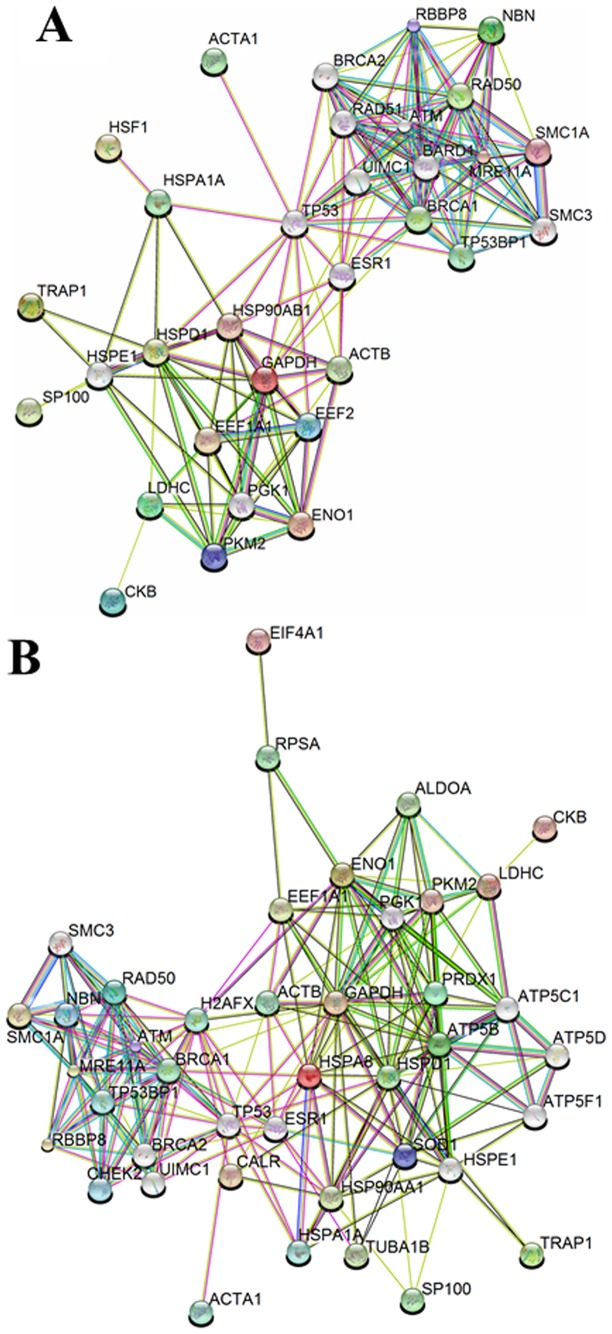
Interactome of the NBN full-length protein. The interactome was gleaned from SDS-PAGE-MS/MS analysis of the streptavidin chromatography eluates obtained from cells expressing the full-length NBN protein (**A**) in control conditions or (**B**) following 2 Gy of X-rays. Colored nodes indicate those proteins that have been identified experimentally in the present study, while grey nodes indicate likely additional interactors that are predicted on the basis of evidences available from the literature. For details, see the text.

Among the NBN full-length protein interactors, the following categories were identified in untreated and treated samples: protein biosynthesis and degradation, nuclear protein import, control of G2/M cell cycle checkpoint, cell growth regulation, transcription regulation, meiosis, DSBs response, activation of the apoptotic process, and oxidative stress response. The identification of several novel NBN interactors contribute to the improvement of the NBN interactome complexity, as gleaned through graphic representation via the software String [49] (Table 1; Fig. 3). It is worthwhile to note that some of the observed interactors were likely to result from a technical bias of the enrichment analytical workflow, as it emerged by assaying the eluates obtained from the enriched protein extracts obtained from empty vector controls (Table 4).

If we exclude false positive results from Table 1 (hits highlighted in yellow), some interactors of the full-length NBN protein are apparently IR-independent (*i.e.*, ENO1, ACTA1, CKB, EEF1A1, LDHC, PKM2/PKLR, and TRAP1) (Table 5).

Several interactors of the full-length NBN protein have been detected only after IR treatment, such as antioxidant enzymes (*i.e.*, PRDX1 and SOD1), chaperones (*i.e.*, HSPA8 and HSP90AA1), calreticulin, metabolic enzymes (*i.e.*, ALDOA and ATP5B), and proteins involved in protein biosynthesis (*i.e.*, EIF4A2 and RPSA) (Table 5).

**Figure 4:**
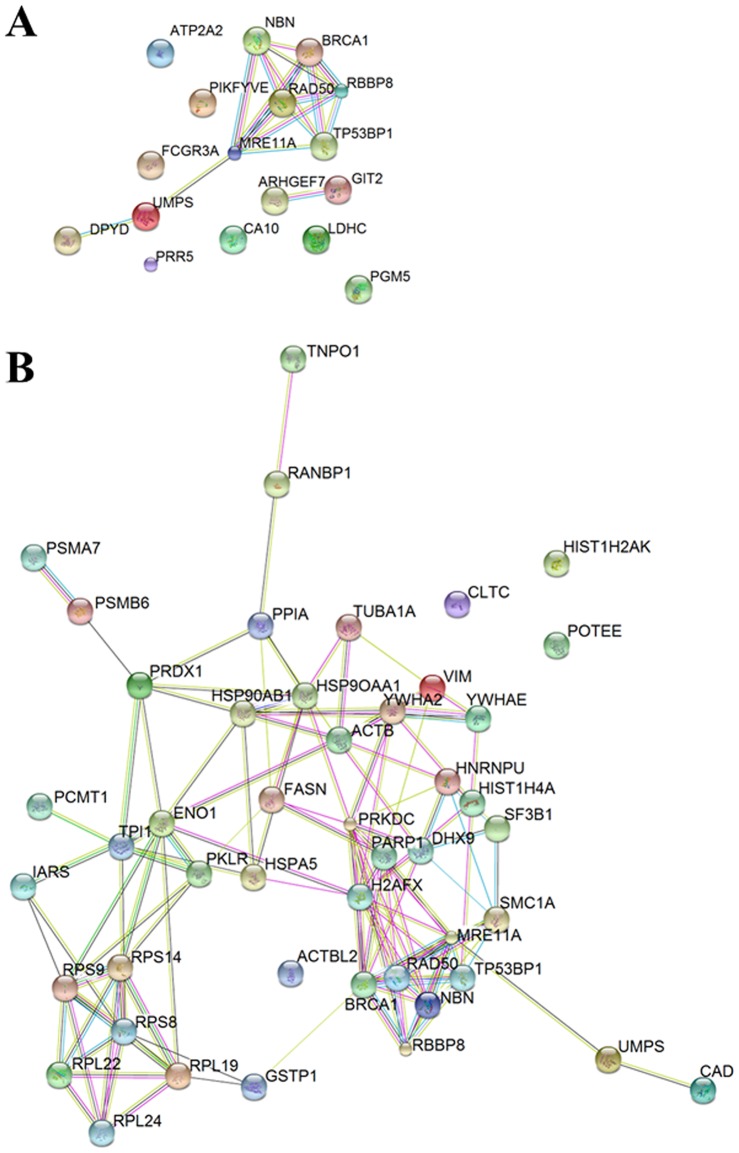
Interactome of the p26 fragment. The interactome was gleaned by merging the results obtained through SDS-PAGE-MS/MS analysis of the streptavidin chromatography eluates obtained from cells expressing the p26 protein fragment (**A**) in control conditions or (**B**) following 2 Gy of X-rays. For details, see the text.

Since PRDX1, an antioxidant that scavenges hydroperoxides, exerts a radioprotective role [54], irradiation can induce PRDX1 expression, thus protecting human cells from IR-induced damage [54]. PRDX1 induction is ATM-dependent, indeed ATM is an important sensor of ROS in human cells [55] and ATM^-/-^ osteoblast are characterized by a reduced induction of PRDX1 after oxidative stress [56]. Therefore, we may speculate that the ATM/NBN axis controls PRDX1 expression, with NBN directly interacting with PRDX1 through the FHA and BRCT1 domains, as suggested by the fact that this protein has been found to be an interactor of p26 after IR (see Tables 2 and 5; Fig. 4).

The identification of SOD1 among the NBN interactors after irradiation supports a role of NBN in the oxidative stress response [57]. Noteworthy, oxidative stress induces cell cycle-dependent MRE11 recruitment, ATM and CHEK2 activation, and histone H2AX phosphorylation, all these events being strictly associated to NBN [58]. Data obtained in conditional null mutant *Nbn* mice have indicated disturbances in redox homeostasis due to impaired DSBs processing [59]. The persistent up-regulation of ROS-related proteins in the liver of irradiated *Nbn* mutant mice may explain the increased DNA damage levels, the chromosomal instability and cancer occurrence in NBS, thus supporting a role of NBN in the repair of ROS-induced damage [59].

The alterations in the chaperone and heat shock protein groups also indicate elevated cellular ROS levels [60]. Chaperones and calreticulin, which is involved in the Ca^2+^-dependent protein folding, have been identified among the NBN interactors after IR treatment. Disturbances in Ca^2+^ homeostasis and protein folding, as well as in the generation of ROS and in the oxidative damage, are essential features of neurodegeneration [61], [62]. Remarkably, one of the main clinical manifestations of NBS are both microcephaly and intellectual disability [3], [40]. Furthermore, both HSPA8 and HSP90AA1 proteins have been demonstrated to be upregulated following genotoxic stress [63], [64] (see below, paragraph “Interactors of the p26 fragment”).

Since the role of NBN in the DNA damage response has been well demonstrated [5], [7], [38], we performed Western blot analysis on the chromatographic eluates to verify the interaction of NBN with proteins playing a key role in the DSBs response pathway. Indeed, since the confidence interval of the mass spectrometry data was set to 0.400 (high confidence), in order to exclude as many false positive interactions as possible, well-known NBN interactors, as well as possible p26 and p70 interactors, might have been excluded. Furthermore, this combined approach allowed us to further analyze the role of the *N*- and *C*-terminal region of NBN in the recruitment of repair proteins on the DSBs.

Immunoblots were performed using the protein eluates obtained from the Strep-tag chromatography (Fig. 2C). Data obtained by Western blot experiments confirmed the NBN full-length protein ability to interact with MRE11, RAD50, ATM, H2AX, CHEK2, BRCA1, SMC1, CtIP, SP100, and 53BP1. These data permitted to verify that the experimental approach allowed the isolation of the DNA repair complex(es) that is(are) known to be assembled in response to the DSBs induction [9].

In particular, NBN full-length interacts with MRE11 and RAD50 (forming the MRN complex) independently from the presence of DNA damage [36], [37], [53], [65]. Similarly, it has been demonstrated that NBN interacts with SMC1 in a BRCA1-dependent fashion, these interactions being fundamental for the proper phosphorylation of both SMC1 and BRCA1 after IR treatment [31]. Remarkably, SMC1 and BRCA1 are involved in a common DNA damage pathway and play a crucial role in the maintenance of chromosomal integrity [31]. Data obtained also confirmed the ability of NBN to interact with the nuclear dots-associated protein SP100 [66], both in basal conditions and after IR treatment. Indeed, the BRCT tandem domains of NBN are responsible for the interaction with SP100 [67], thus allowing their co-localization within the promyelocytic leukemia protein (PML)-nuclear bodies (NB) and in the alternative lengthening of telomere (ALT)-associated PML bodies (APBs) [67]–[69]. Thus, the interaction of NBN with SP100 may be crucial for the genomic stability and for telomere length maintenance [67]. This interaction requires the integrity of the tandem BRCT domains, since the presence of only one BRCT repeat in both the p26 and p70 fragments does not allow the recognition of SP100.

As expected, after DNA damage induction by IR we observed that the NBN full-length protein formed complexes with pSer1981-ATM, γ-H2AX, pThr68-CHEK2, BRCA1, CtIP, SMC1, and 53BP1. Remarkably, these interactions involve the recognition of the phosphorylated forms of these proteins, ad allow the proper DNA damage signaling and cell cycle arrest [23], [25], [31], [50]–[52].

#### Interactors of the p26 fragment

The analysis of p26 interactors highlighted yet undisclosed role of the *N*-terminal region of the NBN protein in cell homeostasis. Indeed, p26 partners can be clustered in three main categories: DSBs repair, anti-oxidant responses, and ribosome joining. Fig. 4 and Table 2 show the interactors of the p26 fragment in untreated (K) and irradiated (RX) transfected cells.

Some metabolic enzymes (*i.e.*, ENO1, LDHC, PRDX1, and PKM2/PKLR) were found among the interactors of both NBN and p26, thus bringing to speculate that the FHA and BRCT1 domains, present in the p26 fragment, mediate these interactions (Table 5). The expression of the p26 fragment in HEK293 resulted in the recruitment of p26-unique IR-dependent partners, as summarized (Tables 2 and 5).

**Figure 5:**
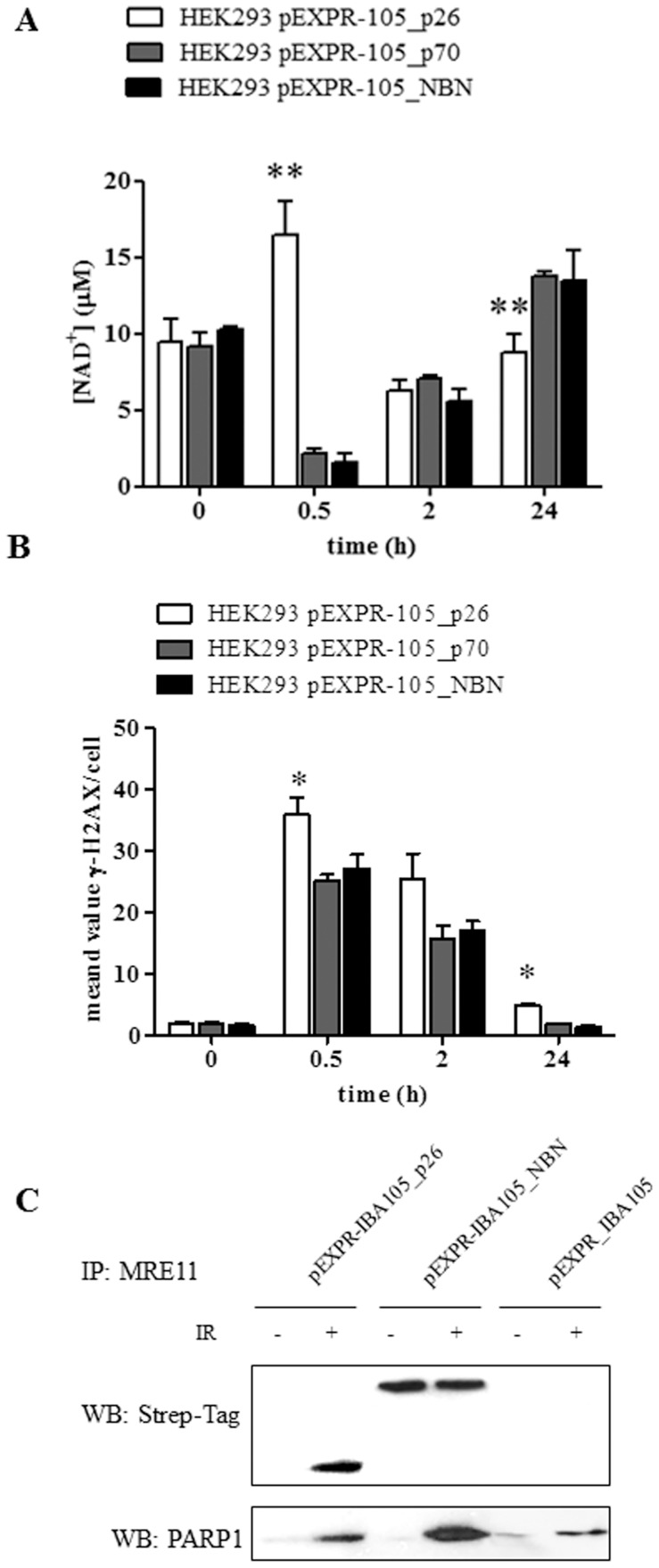
(A) Measurement of NAD^+^ levels in HEK293 transfected cells. Cells transfected with either the full length or the p26 NBN fragment were exposed to 2 Gy of X-rays and harvested after 0.5, 2, and 24 h. The concentration of NAD^+^ was determined using 2×10^5^ transfected cells. Error bars represent the standard deviation from two independent experiments. (**p<0.01; *p<0.05). (**B**) **DSBs repair analysis evaluated by γ-H2AX foci.** HEK293 cells transfected with either the full length or the p26 NBN fragment were exposed to 2 Gy of X-rays and harvested after 0.5, 2, and 24 h. Fixed cells were stained with anti-γ-H2AX, and the number of γ-H2AX foci was counted in 50 cells/experiment, in two repeated experiments. (**p<0.01; *p<0.05). (C) Co-immunoprecipitation experiments aimed at evaluating the interaction of MRE11 with the Strep-tagged proteins and PARP1. HEK293 cells transfected with either the full length or the p26 NBN fragment were exposed to 2 Gy of X-rays and harvested after 0.5 h. Protein eluates were immunoprecipitated with MRE11 and blotted with anti-Strep-tag and anti-PARP1 mouse monoclonal antibodies.

Irradiation of HEK293 cells expressing the p26 fragment promoted the formation of a complex involved in protein biosynthesis (*i.e.*, RPS14, RPS8, RPS9, RPL19, RPL22, and RPL24) and RNA splicing/DNA binding (*i.e.*, HNRNPU, HIST1H2AK, HIST1H4A, SF3B1, DHX9, PARP1, and PRKDC), as gleaned from the protein-protein interaction map in Fig. 4. The formation of these p26-unique IR-dependent complexes might hold potential biological pitfalls in patients suffering of NBS. The binding of PARP1 to damaged DNA, including single-strand breaks (SSBs) and DSBs, through its double zinc finger DNA-binding domain potently activates PARP1 enzymatic activity. Of note, the enzymatic activity of PARPs requires a ready supply of NAD^+^, which is hydrolyzed to produce ADP-ribose units for the PARylation of protein targets [70]. The regulated availability of NAD^+^ may represent a key point of control for PARP1, and the concentration of NAD^+^ has been shown to affect the length of PAR synthesized by PARP1 *in vitro* [71]. NAD^+^ depletion represents PARP1 activity, which is induced by DNA damage and allows its proper signaling and repair [72]–[74]. Here we show that in HEK293 cells over-expressing the p26 fragment a significant increase in NAD^+^ levels was observed at 0.5 h from IR compared to HEK293 cells over-expressing the NBN protein (Fig. 5A). Similarly, it has been reported that NBN-silenced cells, as well as cells established from NBS patients, showed a significant decrease of NAD^+^ depletion following the treatment with either H_2_O_2_ or methyl methanesulfonate, thus demonstrating that PARP1 activity is dependent upon NBN expression [57]. Remarkably, the targets of PARP1 enzymatic activity include PARP1 itself, which is the primary target *in vivo*, core histones, the linker histone H1, and a variety of transcription-related factors that interact with PARP1 [75], [76]. The automodification domain of PARP1 contains several Glu residues that are likely targets for automodification and a BRCT motif that functions in protein-protein interactions [75], [77]. Therefore, we hypothesize that the interaction of p26 with PARP1 after irradiation exerts an inhibitory effect on PARP1 activity, as measured by NAD^+^ levels. Possibly, the interaction of the p26 fragment with PARP1 involves the BRCT domain present in both of them. The BRCT motif is important in protein-protein interaction associated to DNA repair and cell signaling pathways, and several proteins containing BRCT domains interact with specific protein partners by BRCT-BRCT homo- and hetero-interactions. Of note, the BRCT domain of PARP1 is known to mediate the interaction with the BRCT domain of XRCC1, thus allowing an efficient DNA repair [78]–[81]. However, since following irradiation the p70 fragment of NBN undergoes phosphorylation at the Ser278 residue placed within the BRCT2 [41], [82] (see Fig. 1), it might be speculated that this phosphorylation inhibits the interaction of the p70 fragment with PARP1 (see Tables 3 and 5). According to data reported by Digweed's group [83], we speculate that the p26-PARP1 interaction may be responsible for the persistence of ROS, as demonstrated by the NAD^+^ depletion at 24 h from IR in cells over-expressing p26 compared to those over-expressing the full length NBN (Fig. 5A). Noteworthy, this NAD^+^ depletion correlates with the persistence of unrepaired DSBs after 24 h from IR (Fig. 5B). Accordingly with the observation that in NBS patients the truncated p70 NBN fragment is unable to prevent the hyperactivation of PARP1 (with the consequent loss of cellular anti-oxidant capacity) [83], our results suggest that also the p26 NBN fragment may contribute to the NBS phenotype, being responsible for the persistence of high levels of ROS at long time from irradiation. Remarkably, PARP1 has been suggested also as a contributing factor in the pathogenesis of Ataxia telangiectasia, a autosomal recessive disease caused by mutation in ATM [84], [85]. Considering the key role played by NBN in the DSBs sensing and repair, it is interesting to note that among p26-unique IR-dependent interactors a whole subset of proteins involved in DNA/RNA binding and DNA repair (*i.e.*, DHX9, HNRNPU, PARP1, PRKDC, HIST1H2AK, HIST1H4A, and SF3B1) was detected. Notably, DHX9 is an ATP-dependent RNA helicase A that is capable of unwinding double strand DNA and RNA in a 3′ to 5′ direction, and thus functions as a transcriptional activator [86]. Besides, RNA helicase A mediates the association of CBP with RNA polymerase II [87], and the association of BRCA1 to the RNA polymerase II holoenzyme [88], thus influencing DNA transcription. Of note, BRCA1 is a known interacting partner to NBN [31] (see Fig. 2C). On the other hand, the DNA-PK catalytic subunit isoform 1 (PRKDC) is a serine/threonine-protein kinase that acts as a molecular sensor for DNA damage [27], [89]. PRKDC is involved in the non-homologous end joining (NHEJ) repair mechanism required for both DSB repair and the V(D)J recombination, and may also act as a scaffold protein to aid the localization of DNA repair proteins to the site of damage [90].

Finally, among the p26 interactors, the histones HIST1H2AK and HIST1H4A have been identified, as further validated by immunoblot (Fig. 2C). Many histone covalent modifications have been shown to play key regulatory roles in eukaryotic transcription, DNA damage repair, and replication. The chromatin structure has been proposed to make HIST1H4A inaccessible in the absence of DNA damage, with passive relaxation of chromatin at DSBs and the targeted recruitment of histone acetylation, ubiquitination, and chromatin remodeling activities acting to facilitate focal accumulation of 53BP1 [91]–[94]. While the key role of the HIST1H2AK histone in the DSBs response has been well clarified [95], [96], recently new data are highlighting an important role played also by the HIST1H4A [97], [98]. Competitive binding by the hybrid tandem Tudor domains of the JMJD2A and JMJD2B lysine demethylases has been suggested to prevent binding of 53BP1 in the absence of DNA damage, with RNF8-mediated ubiquitination, proteasomal degradation, and rapid depletion of JMJD2A/B from chromatin at damaged sites facilitating focal accumulation of 53BP1 [97]. Recently, it has been demonstrated that transient HIST1H4A deacetylation is an early response to DSBs, which facilitates 53BP1 foci formation, DSB repair by NHEJ, and repression of transcription [98]. HIST1H4A deacetylation may also coordinate with other ATM-and NBN-dependent events to facilitate focal accumulation of 53BP1 at DNA damage sites [99]–[101].

The analysis of p26-unique IR-dependent interacting partners also suggested the yet undisclosed role of this portion of the NBN protein in mediating ribosomal subunit joining (*i.e.*, 40S ribosomal protein S8, S9, and S14, as well as 60S ribosomal protein L19, L22, and L24), in like-fashion to the Shwachman-Diamond Syndrome protein (SBDS) [102], [103]. However, it is also important to stress that several ribosomal proteins have extra-ribosomal functions, including replication and DNA repair. Therefore, either mutations in ribosomal proteins or alterations to their interacting partners may have effects that are independent of the protein translation machinery [104].

Interestingly, when looking at the interactors of the p26 fragment, we observed the presence of proteins able to bind, either directly or indirectly, the FHA and BRCT domains (Fig. 2C). While some authors suggested that tandem BRCT domains of NBN mediate the interaction with phosphorylated MDC1 [95], [105], several experimental evidences indicate that NBN is able to bind directly γ-H2AX, the FHA and tandem BRCT domains of NBN having a crucial role in mediating this interaction [15], [53], [106], [107]. Therefore, it was not surprising that our data indicate that only the p26 fragment of NBN was able to interact with γ-H2AX. Remarkably, it has been previously demonstrated that the p26 and p70 NBN fragments were able to co-immunoprecipitate with γ-H2AX [53]. These evidences brought to the hypothesis that the BRCT domains present in p26 and p70 are involved in the dimerization of the two NBN fragments, thus recreating the pocket that allows the interaction with γ-H2AX [15], [53], [108]. Remarkably, our results indicate that while the FHA and BRCT1 domains present in the p26 fragment seem sufficient to bind (either directly or indirectly) γ-H2AX, the BRCT2 domain present in the p70 fragment does not. Indeed, differently from NBS cells, HEK293 cells were transfected either with the p26 or the p70 fragment, and H2AX has been identified among the interactors of the p26 fragment upon irradiation, but not among the interactors of the p70 fragment.

Specifically, γ-H2AX [53], [106], [107], BRCA1 [23], [30], [31], and CtIP [13], [21]–[27] were found among the interactors of the p26 fragment, thus confirming that the FHA and the BRCT1 domains maintain the capability to interact with proteins involved in the DNA damage response (Fig. 2C). Remarkably, our data agree with previous reports indicating that the interaction of the MRN complex with BRCA1 is ATM- and NBN-dependent [31]. In particular, IR enhances ATM binding to BRCA1 [33]. However, both the *N*- and *C*-terminal domains of NBN seem to be required for the proper interaction with BRCA1 [31], as further supported by our data indicating that both the p26 and p70 fragments bind BRCA1. Noteworthy, looking at the p26 interactors, also MRE11 and RAD50 were detected by Western blot analysis. This result appears surprising since p26 lacks both the MRE11- and ATM-binding motifs. Indeed, the absence of both the MRE11-interaction motifs identified in NBN (*i.e.*, the interaction motif 2 comprised between amino acids 638–662, and the interaction motif 1 comprised between amino acids 682–693) [35], prompted us to further investigate why MRE11 was identified among the p26 NBN interactors. Of note, MRE11 has been described to interact with PARP1 at sites of DSBs, and this interaction is required for rapid accumulation of MRE11 protein, and in turn of the MRN complex, at DSBs [72]. Furthermore, in mammalian cells the presence of PARP1 and MRE11 proteins in a functional complex has been described as an important mechanism in resolving DNA lesions [108]. Remarkably, after irradiation, we observed that p26 interacts with PARP1 (see Table 2 and Fig. 2C), and, in turn, MRE11 interacts with both the Strep-tagged p26 NBN fragment and PARP1 (Fig. 5C). Of note, while MRE11 co-immunoprecipitated with the Strep-tagged full length NBN both in basal conditions and after irradiation, the interaction with the p26-tagged fragment, as well as with PARP1, took place only after DSBs induction (Fig. 5C). At the light of these results, our hypothesis is that the observed interaction between the p26 NBN fragment and MRE11 is mediated by the PARP1 common interactor.

Hsp90 chaperone machinery ensures the function of proteins important for DNA repair, recombination, and chromosome segregation [109]. It has been recently demonstrated that BRCA1 and Hsp90 cooperate in homologous and non-homologous DSBs repair, as well as in the G2/M checkpoint activation [110]. Hsp90 is known to associate with the MRN complex, although the molecular mechanism is yet unknown [111]. In particular, Hsp90 seems to associate with NBN to facilitate the NBN/ATM interaction, and the use of the Hsp90 inhibitor 17-(dimethylaminoethylamino)-17-demethoxygeldanamycin (17DMAG) reduces the interaction between NBN and ATM, although no degradation of the MRN complex has been detected. The diminished radiation-induced activation of ATM in 17DMAG-treated cells seems to be the result of a compromised function of the MRN complex [111]. Results obtained by mass-spectrometry indicate Hsp90 as an interactor of both the NBN full length and the p26 fragment after IR. This result has further validated by immunoblot experiments (Fig. 2C). Of note, Hsp90 does not interact with the 70 kDa NBN fragment (Fig. 2C). These results strongly suggest that at least the *N*-terminal region of NBN is involved in the recruitment of Hsp90 in the MRN complex in response to the IR-induced damage. Overall, data from literature support our mass spectrometry data, indicating that Hsp90 may somehow contribute to the maintenance of the genome stability, although the molecular mechanism needs to be further clarified.

As already known, the phosphorylated form of CtIP is recruited to the DNA damaged site by direct interaction with the FHA domain of NBN [13], [23], as confirmed by the identification of this protein among the p26 interactors (Fig. 2C). However, the absence of ATM, CHEK2, and SMC1 among the interactors of p26 and p70 fragments after IR impairs the proper DNA damage signaling, thus allowing the proliferation of unrepaired or misrepaired cells.

#### Interactors of the p70 fragment

**Figure 6:**
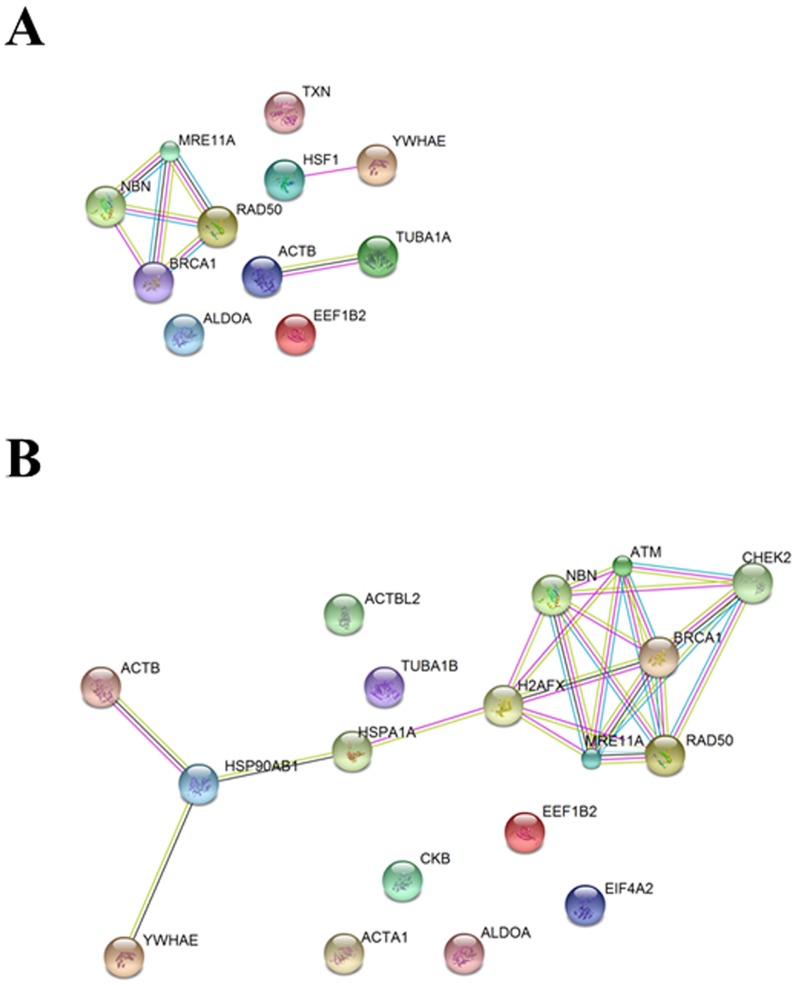
Interactome of the p70 fragment. The interactome was gleaned by merging the results obtained through SDS-PAGE-MS/MS analysis of the streptavidin chromatography eluates obtained from cells expressing the p70 protein fragment (**A**) in control conditions or (**B**) following 2 Gy of X-rays. For details, see the text.

The p70 protein fragment showed a limited number of shared interactors with respect to the full-length NBN protein (Fig. 6). The p70 interactors can be clustered in two main categories: helicase activity and regulation of protein import into nucleus. Among these interactors ALDOA, CKB, and EIF4A2 were identified (Tables 3 and 5). The reduced complexity of the p70 interactome after irradiation allow hypothesizing that the single BRCT2 domain and the MRE11- and ATM-binding motifs are not involved in the recognition of the full-length NBN interactors.

Very low amounts of p70 in lymphoblastoid B-cell lines correlate with B-cell lymphoma development in NBS patients [42]. Similarly, the disease-causing Arg215Trp NBN mutation is associated with low levels of the NBN mutated protein in cell lines, and with a significant predisposition to cancer development in the heterozygous carriers [112], [113]. Therefore, it has been postulated that even loss of one *NBN* allele increases cancer risk [114], this hypothesis being in line with the concept of DNA repair is a primary cancer avoidance pathway, in which repair enzyme levels are critical for lifetime cancer risk [115]. Remarkably, p70 variation among NBS patients is not determined at the transcription level, but rather by protein stability. Indeed, *in vitro* inhibition of the proteasome increases cellular levels of p70 [116]. Lastly, the irradiation of cells transfected with p26 caused its interaction with two proteasomal components (*i.e.*, proteasome HSPC and proteasome subunit Y). This might suggests that p70 clearance might be driven by p26, though further studies are mandatory.

As expected from the presence of the MRE11-binding motif [35]–[37], [65], [117]–[119], MRE11 and RAD50 were identified among the p70 interactors (Fig. 2C). Notably, this represents an important clinical feature, since patients whose cells display high levels of the truncated p70 protein are at a low risk for lymphoma than those patients with low levels of p70 in their cells [42]. Furthermore, ATM [10], [16]–[20], [120], CHK2 [50], [51], [52], [65] and BRCA1 [23], [30], [31] bind directly or indirectly to the ATM-binding motif present at the *C*-terminus of the 70 kDa fragment (Fig. 2C).

## Conclusions

Several relevant clinical and experimental evidence prompted us to evaluate the residual activity of p26 and p70 fragments through a proteomic characterization of the interactors, both in basal condition and after irradiation.

1. The 657del5 mutation is a hypomorphic mutation causing only a partial loss of the *NBN* gene function. Remarkably, in mice null mutation of NBN leads to embryonic lethality [121], [122] and only animals with hypomorphic mutations survive past early embryogenesis [123], [124]. Remarkably, transduction of inducible knock-out mice cells with a cDNA containing the 657del5 mutation, rescued cells from death in culture [2]. This clearly indicate that the 26 and 70 kDa fragments arising from the 657del5 mutation retain a residual function, supporting the notion that NBS patients survive owing to the expression of these fragments [2].

2. NBS patients displaying high intracellular levels of the p70 truncated protein are at lower risk for lymphoma than NBS patients expressing low levels of p70 [42].

3. Cells established from NBS patients survive following irradiation, but retain γ-H2AX foci due to a subtle DSBs repair defect, albeit at a lower level compared to Ataxia telangiectasia (AT) lymphocytes (characterized by the absence of the ATM protein) [95], [125], [126]. The slightness of the defect observed in NBS cells compared to AT ones might reflect the expression of the p26 and p70 NBN proteins that, maintaining residual protein activity may contribute, although with a reduced efficiency, to the genome integrity. Indeed, even if in NBS cells the defect of the overall rejoining of DSBs is subtle, a more substantial defect in the correct rejoining of DSBs has been demonstrated [126].

4. Though NBS is a recessive disease and one would not expect any cellular feature or clinical symptom, a growing number of papers report higher spontaneous and induced chromosome instability and an increased incidence of tumors among NBS carriers [127]–[129]. Remarkably, Nbn+/− mice showed a significantly increased occurrence of spontaneous solid tumors in addition to lymphoma. Moreover, IR dramatically increased cancer formation in Nbn^+/-^ mice, especially thyroid tumors. These data provide a clear relationship between NBN heterozygosity, radiation sensitivity and increased cancer risk. Interestingly, examination of the tumors gave no evidence for loss or mutation of the wild-type allele, suggesting that haploinsufficiency is the presumed pathogenic mechanism [121]. In human heterozygotes, the existence of two truncated proteins produced by alternative translation induced by the 657del5 mutation is compatible with a dominant negative mechanism [41], [129].

The present study reports for the first time a proteomic analysis of the interactors of both the full-length NBN protein and of its fragments arising from the 657del5 founder mutation, responsible for the development of NBS. The application of an unsupervised proteomics approach suggested previously unreported protein interacting partners to the NBN protein and to the p26 and p70 fragments (possessing the FHA/BRCT1 domains, and the BRCT2 domain/MRE11- and ATM- recognition domain, respectively). The phenotype caused by the expression of two proteins normally absent within a cell may arise from a gain-of-function that causes the pathological phenotype deriving from their expression at the homozygous status in NBS patients. Furthermore, since the tandem BRCT domains are the major mediators of phosphorylation-dependent protein-protein interactions, the loss of their integrity is expected to affect the interaction with a number of proteins. Our results revealed that approximately the 30% of the interactors are shared by the full length NBN and the p26 fragment, while approximately the 41% of the interactors are shared by the full length NBN and the p70 fragment. These results highlight several relevant aspects: (*i*) the two fragments possess a residual protein activity; (*ii*) the disruption of NBN allows a partial maintenance of the wild-type functions; and (*iii*) each fragment acquire a gain-of-function determined by the domains and motifs present.

In particular, results obtained shed light on new possible roles of NBN and of its p26 fragment in ROS scavenging, in the DNA damage response, and in protein folding and degradation. Furthermore, the *N*-terminus of NBN might enroll protein partners involved in the DSBs repair and in the anti-oxidant response. Since some of the newly identified interactors of the p26 and p70 fragments have not been found to interact with the full-length NBN, this suggests that these interactions may somehow contribute to the key biological phenomena underpinning the Nijmegen breakage syndrome. Overall, further studies will be necessary to clarify the biological significance of the newly identified interactors of NBN in cell homeostasis and in the DNA damage response.
